# Empirical Evidence Supporting Frequent Cryptic Speciation in Epiphyllous Liverworts: A Case Study of the *Cololejeunea lanciloba* Complex

**DOI:** 10.1371/journal.pone.0084124

**Published:** 2013-12-18

**Authors:** Ying Yu, Jochen Heinrichs, Rui-Liang Zhu, Harald Schneider

**Affiliations:** 1 Department of Biology, East China Normal University, Shanghai, China; 2 Department of Life Science, Natural History Museum, London, United Kingdom; 3 Albrecht-von-Haller-Institut für Pflanzenwissenschaften, Georg-August-Universität, Göttingen, Germany; 4 Department of Biology, Systematic Botany and Mycology, University of Munich (LMU), Munich, Germany; 5 Shanghai Key Lab for Urban Ecological Processes and Eco-Restoration, East China Normal University, Shanghai, China; 6 The State Key Laboratory of Systematic and Evolutionary Botany, Institute of Botany, Chinese Academy of Sciences, Beijing, China,; Wuhan Botanical Garden, Chinese Academy of Sciences, Wuhan, China, China

## Abstract

Cryptic species are frequently recovered in plant lineages, and considered an important cause for divergent of morphological disparity and species diversity. The identification of cryptic species has important implications for the assessment of conservation needs of species aggregates. The mechanisms and processes of the origin of cryptic species diversity are still poorly understand based on the lack of studies especially in context of environment factors. Here we explored evidence for cryptic species within the epiphyllous liverworts *Cololejeunea lanciloba* complex based on two loci, the plastid *trnL*-*F* region and the nuclear ribosomal ITS region. Several analytic approaches were employed to delimit species based on DNA sequence variation including phylogenetic reconstruction, statistical parsimony networks analysis and two recently introduced species delimitation criteria: Rosenberg’s reciprocal monophyly and Rodrigo’s randomly distinct. We found evidence for thirteen genetically distinct putative species, each consisting of more than one haplotype, rather than four morphologically-circumscribed species. The results implied that the highly conserved phenotypes are not congruent with the genetic differentiation, contributing to incorrect assessments of the biodiversity of epiphyllous liverworts. We hypothesize that evolution of cryptic species recovered may be caused by selection of traits critical to the survival in epiphyllous habitats combined with limited developmental options designed in the small body.

## Introduction

Cryptic species, i.e. biological entities with reproductive isolation and/or genetic divergence without recognizable morphological disparity [1], are a major challenge to biodiversity research. Despite our limited understanding of the processes, some evidence indicates that the origin of some or many cryptic species may coincide with the adaptation to extreme habitats [1,2]. This hypothesis is consistent with the observation of large number of cryptic species of animals occurring in extreme habitats [3-5] and cryptic land plant species growing either in aquatic or epiphyllous habitats [6-9] or in habitats with considerable desiccation stress [10]. However, this hypothesis may be correct for some but very unlikely for all cases of cryptic species diversity as indicated by cryptic species of animal and plant lineages that don’t occur in extreme habitats [11-14]. Little attention has been so far given to the origin of cryptic species in liverworts, particular for the diversity of cryptic species in extreme habitat besides documenting its putative origins. Here, we explored the hypothesis of cryptic species in the case of derived leafy liverworts growing in extreme environment by an attempt to provide some insights into the correlations of restricted “ecospace” and origin of cryptic species.

The liverwort genus *Cololejeunea* is a well-suited model to infer the evolution of cryptic species in extreme environments. The genus represents not only one of the most species rich genera of liverworts with more than 400 currently accepted morphologically-typologically circumscribed species (MTSs) (according to the “Early Land Plant Today” project) , but also a predominate component of epiphyllous liverworts-growing preferentially at the surface of leaves of vascular plants (= epiphylly) [15]. The epiphyllous habitat provides substantial challenges to the survival of liverworts, such as limited access to water and nutrients, and the ephemeral conditions of the substrate. Such harsh conditions restricted the successful colonization of this habitat to only a few lineages of leafy liverworts, such as a few derived genera of Lejeuneaceae subfamily Lejeuneoideae [16]. This study was designed to explore evidence for cryptic speciation in a well-defined epiphyllous group, the *Cololejeunea lanciloba* complex [15,17], with focus on *Cololejeunea lanciloba* Steph., *C. planissima* (Mitt.) Abeyw., *C. latilobula* (Herzog) Tixier and *C. yakusimensis* (S.Hatt.) Mizut. These four species represent common pantropic epiphyllous liverworts with a distribution center of Asian, and were critically studied [15,17-19]. Some characters such as sexuality, range of hyaline cell on leaf margin and lobule shape have traditionally been treated as diagnostic traits to distinct these species (Table 1). Regardless of unambiguous circumscription of these taxon based on morpholgy, the monophyly of each species was not supported with exception of *C. yakusimensis* in a recent molecular study, albeit samples were limited [20]. We restricted our study to Chinese and neigbour regions occurrence of this complex and included only two species occurring outside of China. This restriction was due to the lack of reliable assessment of the total number of species of the complex outside of these regions.

**Table 1:** Summary of morphological variation of four species studied.

Characters	*C. lanciloba*	*C. latilobula*	*C.planissima*	*C. yakusimensis*
Sexuality*	autoicous	autoicous	autoicous	synoicous or paroicous
Oil body	5-12 per median cell	5-16 per median cell	5-11 per median cell	3-9 per median cell
Hyaline cells*	present throughout leaf margin	only on antical margin	only on antical margin	only on antical margin
Lobule*	narrowly ligulate or triangular-lanceolate with 1-2 additional teeth	usually ligulate (rarely triangular) without additional teeth at margin	mostly ovate or triangular with a tooth at proximal margin if lobule lanceolate or ligulate) and/or 2-3 teeth at apex of lobule if lobule ovate to triangular-ovate	usually ligulate (rarely triangular) without additional teeth at margin
Hyaline papilla	subapical (apical)	apical	proximal or apical	apical
Stylus	unicellular (1-2 celled occasionally)	1-celled	1-2 celled	unicellular
Cuticle	nearly smooth to finely punctate	punctate	nearly smooth to finely punctate	nearly smooth to finely punctate
Gemmae	24-30 celled	26-celled	26-38 celled	22-30 celled

^(*)^ represents key morphological characters commonly used for classification of these taxa. The literatures consulted for morphological data collection here included Mizutani [35], Tixier [36], Zhu & So [15] and Daniels, Kariyappa and Daniels [18].

In the recent years, delimitation of species using molecular evolution was addressed in a rapidly increasing number of studies [21-23]. These studies challenged the previous practice that species boundaries were delimited hitherto mainly on phenotypic evidence of organism. To achieve reliable delimitation of species using molecular data, several promising methods have been introduced to identify species based on phylogenetic hypotheses, namely Kimura 2-parameter distances (K2P) [24], Rosenberg’s reciprocal monophyly P(AB) [25], Rodrigo’s P(RD) measures [26], General mixed Yule coalescent (GYMC) [27], and Bayesian Phylogenetics & Phylogeography (BP&P) [28]. These methods have obtained varying degree of success measured as recovery of putative species of animals [29-32], but were rarely applied to plant lineages. Among these methods, the criteria Rosenberg’s reciprocal monophyly P(AB) and Rodrigo’s randomly distinct P(RD) based on the coalescent distinctiveness, have been indicated more sensitive to detect taxonomic distinctiveness than some measures, such as K2P distances and clade support [33]. Some of these methods even work on single locus where other methods work best with multilocus information such as some coalescent hypothesis based methods and most of the clustering methods [27]. Considering above arguments, we employ the criteria P(AB) and P(RD) plus an iterative ‘tip-to-root’ approach described in Boykin et al. [33] for species delimitation to assess taxonomic distinctiveness of the four MTSs of interest. Furthermore, we used a statistic parsimony analyses considered as a fast and effectively method to detect putatively undescribed species [34].

We tested the hypothesis that the *Cololejeunea lanciloba* complex includes more than above four taxa as recognized in most recent taxonomic treatments [15]. Additionally, we explored evidence for coincidence of ecological preference to the extreme conditions of the epiphyllous habitat and the formation of cryptic species. The later process may also involve the parallel evolution of similar phenotypes. To achieve this, we sampled a total of 62 accessions and generated sequences of two loci, one located in the nuclear and one in the plastid genome. 

## Materials and Methods

### Ethics statement

 The plant sampling of this study includes 21 accessions derived from Yu et al. [20] and 41 accessions borrowed from herbariums of East China Normal University (HSNU) and Royal Botanical Garden Edinburgh (E) (Table S1). No specific permission for field work was needed for this study and the sampling was carried out without destroying local populations.

### Molecular phylogeny

**Figure 1:**
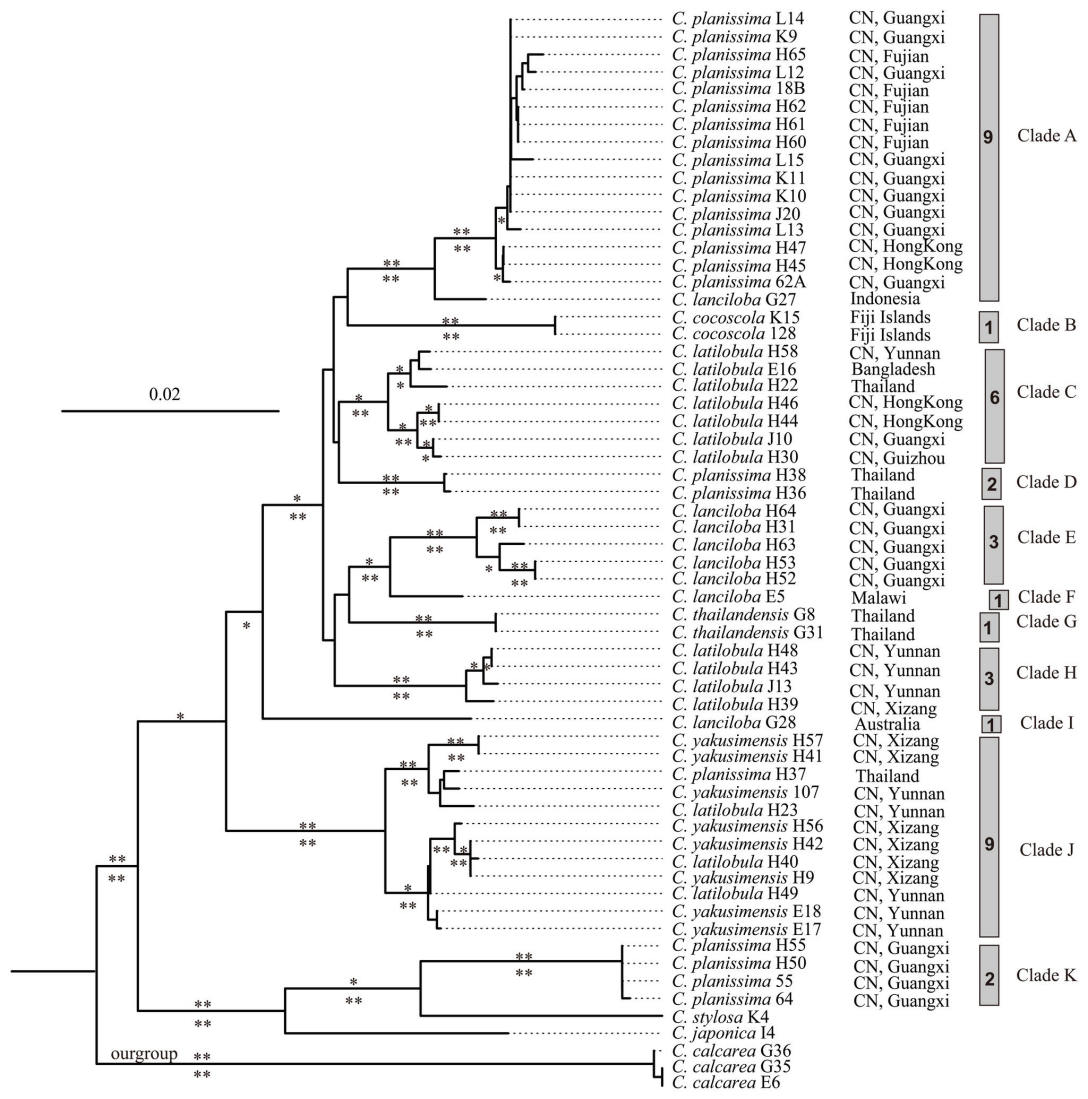
The most likely phylogram obtained in a maximum likelihood analysis of the combined dataset. Bootstrap values ≥ 95% (** = 100%, * = 95-99%) were plotted above branches and posterior support values ≥ 0.95 (**=1.00, *=0.95-0.99) below branches. Shown branch lengths correspond to the estimated substitution events (see bar). Vertical bars on the right side of the figure mark specimens that were recovered as part of a single network in statistical parsimony analyses of the combined dataset. The numbers correspond to the number of haplotypes per network. Letters on the right side correspond to molecular operational taxonomical units (MOTUs) discussed in the text. The amplification numbers and region are given for each specimen.

We sampled a total of 59 accessions representing the ingroup (Table S1), including *Cololejeunea lanciloba* (8 accessions), *C. planissima* (23 accessions.), *C. latilobula* (14 accessions), and *C. yakusimensis* (8 accessions). In addition, the ingroup also comprised two accessions each of two other members of *C. lanciloba* complex (*C. cocoscola* and *C. thailandensis*) and one accession each of two relatively closely related taxa (*C. japonica* (Schiffn.) Mizut. and *C. stylosa* (Steph.)A. Evans).The outgroup comprising three accessions of *Cololejeunea calcarea* (Libert.) Schiffn. was chosen based on the phylogeny of *Cololejeunea* [20]. The key morphological data of four MTSs of interest was assembled based on the description from literatures [15,35,36] and observations on available herbarium specimens (see Table 1). These studies should aim to include DNA sequence data of all type specimens as far as the preservation of the specimens enable DNA amplification. Each specimen studied was carefully identified using morphological evidence (see Table 1) before and after phylogenetic analyses. The chloroplast *trnL–F* and nuclear regions nrITS were obtained for all 62 accessions as described [20] or by downloading from GenBank (voucher information see Table S1). Sequences were aligned using PhyDE v0.9971 (http://www.phyde.de/). Ambiguous positions, positions for which at least two equally probable alignments were obtained, were identified visually and removed from alignment for subsequent analyses. Exclusion of ambiguous regions, the dataset was reduced from 1620bp to 1464bp (ca. 90% of the original matrix). The data matrices and a single tree of Maximum likelihood analyses (Figure 1) are deposited on TreeBase (URL: http://purl.org/phylo/treebase/phylows/study/TB2:S14897). All phylogenetic and network analyses were carried out for both regions independently and the combined dataset.

Maximum likelihood analyses (ML) for separate regions and a combined dataset were conducted with PhyML 3.0 [37] as implemented in the PhyML plugin of Geneious 5.5.6 (http://www.geneious.com/). The optimal models of evolution were selected in jModeltest 0.1.1 [38] using Akaike Information Criterion (AIC) and hierarchical likelihood ratio test (hLRT): TIM + I for *trnL–F*, TrN + I + G for nrITS, and TrNef + G for the combined dataset. Bootstrap values for ML analyses were obtained via 200 bootstrap replicates in PhyML using the above fit model and parameters as in the optimal tree search. Combinability of the plastid and nuclear regions were inferred by comparing the bootstrap consensus trees, considering the 70% bootstrap value criterion as indicator of topological heterogeneity [39]. Bayesian inference of phylogeny for the combined dataset was performed using MrBayes 3.2.1 [40], and a partitioning into chloroplast region (*trnL–F*) and nuclear region (nrITS). Bayesian search was carried out with the GTR model implemented, parameter values inferred simultaneously with tree searcher, runs starting with a random tree, unlinked rates. The model was selected using the Bayesian Information Criteria (BIC) implemented in jModeltest. Four Markov chain Monte Carlo runs consisted of five million generations with a sampling frequency of one every 1,000th generation. The convergence of runs and burn-in phase were estimated in Tracer v 1.4.4 (http;//beast.bio.ac.uk/Tracer). The first 500,000 samples were discarded as burn-in samples. Bayesian posterior probabilities (BP-PP) were calculated as majority consensus tree of the remaining 4,500,000 trees after discarding the trees sampled within the burn-in phase. 

ALTER (http://sing.ei.uvigo.es/ALTER/) was used to transform the matrix into the format required for TCS and to identify the number of haplotypes. Plausible connections between haplotypes were reconstructed using statistical parsimony approach (SPNA) with a 95% connection limit as implemented in TCS v1.21 [41,42]. To minimize the number of ambiguously inferred haplotypes, indels and one sample with missing data, *Cololejeunea planissima* L13, were excluded from the alignment for SPNA. 

### Species distinctiveness measures

Rosenberg’s reciprocal monophyly P(AB) and Rodrigo’s (P(RD) measures were calculated using the species delimitation plugin [43] for Geneious (www.biomatters.com). Two approaches were applied to investigate the species distinctiveness. Firstly, we tested distinctiveness by P(AB) and P(RD) for seven molecular operational taxonomy units (MOTUs) out of the eleven MOTUs that were selected based on clade support and the independence of network on 95% connection limit in SPNA. If two sister clades both have branch bootstrap ≥ 95% and involved in individual network in SPNA, we suggested that these two lineages are isolated and distinct. Four MOTUs, each including a single haplotype prohibiting the calculation of the two criteria, were excluded. Secondly, the taxonomic distinctiveness was assessed with the iterative ‘tip-to-root’ approach [33] on phylogeny without any predefined MOTUs. In the “tip-to-root” process, we assigned a unique number for each clade (e.g. 1–1) comprising two or more individuals in the phylogeny of *Cololejeunea lanciloba* complex. Every group or clade was tested against its sister group to assess the significant of distinctiveness according to P(AB) and P(RD). This process starts at the tips of the tree and works along the branches. 

## Results

### Phylogenetic and statistics parsimony network analyses

The combined dataset included 1,464 (1,062 nrITS, 402 *trnL–F*) characters of which 51 (42 nrITS, 9 *trnL*–*F*) were autapomorphic, and 285 (239 nrITS, 46 *trnL–F*) parsimonious informative. No evidence for topological incongruence was recovered between the two regions (Figure S1). Identical topologies were found in the consensus tree of the Bayesian analyses of the combined dataset with a partition in two regions (mean likelihood of –InL = 5,453.208) and the most likely tree found in the maximum likelihood analyses (–InL = 5,606.879). All clades based on the combined dataset were also recovered in the phylogeny based on the nrITS. A majority of deep nodes lacked support in the phylogenetic hypothesis based solely on the *trnL–F* region (Figure S1).

 The four MTSs investigated were not resolved as monophyletic groups (Figure 1). Specimens identified as *Cololejeunea planissima* were nested in four clades: A, D, J and K, whereas those identified as *C. latilobula* were nested in clade C, H and J. The polyphyly of *C. lanciloba* was attributed to two accessions, one from Australia (Clade I) and another one from Indonesia (Clade F), distinct from clade E comprising of Chinese *C. lanciloba* specimens (Figure 1). Accessions of *C. yakusimenis* were nested in clade J, which also included samples identified as *C. latilobula* and *C. planissima*. 

A total of 20, 36 and 38 haplotypes were found within the ingroup based on *trnL–F*, nrITS and the combined data set respectively. SPNA analyses recovered 7 independent networks and 4 isolated single haplotypes based on the combined dataset (Figure S2). In the phylogenetic analyses, the clades corresponding to these independent networks obtained high bootstrap values (ML-BP ≥ 95%) and posterior probabilities (BP-PP ≥ 0.95) (Figure 1; Table 2). 

**Table 2:** Summary of the accumulated evidence supporting molecular operational taxonomical units (MOTUs) A to K.

MOTUs	SPNA (Ind.)	Bootstrap support (%)/ posterior probability			Proposed species (P(AB) & P(RD))		
		*trnL-F*	ITS	*trnL-F*+ITS	*trnL-F*	ITS	*trnL-F*+ITS
Clade A	Yes	90/1.0	100/1.0	100/1.0	Clade A (+)	Clade A (Clade 1-8, *C. lanciloba* G27)	Clade A (clade 1-9, *C. lanciloba* G27)
						Clade 1-9 (+)	Clade 1-8 (+)
Clade B	(1)	−/0.98	100/1.0	100/1.0	Untested	Untested	Untested
Clade C	Yes	−/0.99	100/1.0	99/1.0	Clade C	Clade C (Clade 1-13)	Clade C (Clade 1-13)
						Clade 1-10 (+)	Clade 1-10 (+)
						Clade 1-11 (+)	Clade 1-11 (+)
Clade D	Yes	−/−	100/1.0	100/1.0	−	Clade D	Clade D (+)
Clade E	Yes	95/1.0	99/1.0	100/1.0	Clade E (Clade 1-19)	Clade E (Clade 1-19)	Clade 1-18 (+)
					Clade 1-18 (+)	Clade 1-18 (+)	Clade 1-19 (+)
Clade F	(1)	−/−	−/−	−/−	Untested	Untested	Untested
Clade G	(1)	73/0.98	100/1.0	100/1.0	Untested	Untested	Untested
Clade H	Yes	87/−	100/1.0	100/1.0	Clade H	Clade H	Clade H
Clade I	(1)	−/−	−/−	−/−	Untested	Untested	Untested
Clade J	Yes	84/0.87	100/1.0	100/1.0	Clade 2-5	Clade J (Clade 2-1, 2-6)	Clade J (Clade 2-1)
						Clade 2-2 (+)	Clade 2-2 (+)
							Clade 2-6 (+)
Clade K	Yes	93/0.99	100/1.0	100/1.0	Clade K (+)	Clade K (+)	Clade K (+)

Values can only be shown for MOTUs represented with more than one haplotype. −: values are unavailable. +: genetic distinctiveness at significant level as P(AB) < 10^-5^ or P(RD) ≤ 0.05. Single- haplotype clades are indicated by number 1. The second column showed the independence of network.

### Species distinctiveness measures

Seven predefined MOTUs (clade A, C–E, H, J and K) were tested using P(AB) and P(RD) species distinctiveness measures. Under strict criteria, the mean probabilities (P ID (strict)) ranged from 0.55 (0.40 to 0.70) for clade D to 0.94 (0.89 to 0.99) for clade A (Table S2). When using more liberal criteria, the mean probabilities for all MOTUs investigated were above 90% (Table S2). Clade D and K have P(RD) values < 0.05, whereas clade A and clade J have P(AB) values < 10^-5^. The clades D, E and H did not show genetic distinctiveness at significant level of P (AD) < 0.05 or P(AB) < 10^-5^ despite they were found with support values of ML–BP = 100% and BP–PP =1.0 (Figure 1; Table S2). 

The ‘tip-to-root’ approach recovered six additional MOTUs nested within the predefined seven MOTUs (Figure S3; Table S3, Table 2). These six additional MOTUs were: one each in clade A and E, and two each in clade C and J (Table 2). The results of species delimitation based on nrITS were similar with those based on the combined dataset (Table 2). A total of five additional MOTUs were revealed based on ITS: one each in clade A, E and J, and two in clade C (Table 2). The *trnL–F* data supported total seven MOTUs: one each in clade A, C, H, J and K, and two in clade E excluding untested clades (Figure 1; Table 2). 

 Overall, the analyses recovered evidence for distinct 13 MOTUs using P(AB), P(RD), clade support and SPNA, after excluding untested MOTUs comprising a single haplotype: one each in clade D, H and K, two each in clade A and E, and three each in clade C and J (Table 2).

## Discussion

### Cryptic species within *Cololejeunea lanciloba* complex

Our results revealed that the monophyly of each MTS of interest were not supported by genotypic evidence. This conclusion was supported from both the nuclear and the plastid genome. In turn, we identified up to thirteen genetic distinct MOTUs among four MTSs investigated using statistical criterias P(AB) and P(RD). Specimens of *Cololejeunea planissima*, *C. latilobula* and *C. lanciloba* were nested in more than one clade separately, suggesting the discordance of phenotype and genotype. This conflict between phenotype and genotype was evident in particular for *Cololejeunea planissima* (clade 1-9, 1-8, D, K) and *C. latilobula* (clade1-13, 1-10, 1-11, H). The recovered topology indicated the occurrence of cryptic species. All accessions of *C. yakusimensis* together with some accessions from *C. latilobula* and *C. planissima* were nested in one clade J (Figure 1). These results suggested the occurrence of morphological plasticity. 

Incongruence between morphological and molecular evidence has been reported in various plant lineages [44,45], including mosses and liverworts [46-49]. Several mechanisms considered to create these conflicts have been widely recognized including the occurrence of cryptic speciation. The origin of some cryptic species cases coincide either with extrinsic factors, e.g., ecological selection, or with inherited constraints, such as development constraints and functional constraints [2], whereas some cryptic species are the result of hybridization [50], and/ or introgression [51] and/or chromosome doubling [52]. 

Natural selection was considered to foster cryptic species via direct and/or indirect selection on phenotype and/or reproductive traits [6,53,54]. In our case, epiphyllous liverworts likely experience strong selection on morphological characters that are critical for the survival of these plants in harsh condition-at the surface of living leaves. The putatively adaptive characters of epiphyllous liverworts include small size of the gametophyte body, inflated leaf lobules and/or presence of papillosae cells for the retention of water, appressed stems and secondary rhizoid discs supporting attachment to the smooth leaf surface, substantial asexual production and inbreeding supporting effective colonization of ephemeral substrates [55,56]. Observations on rheotypic relatives of epiphyllous liverworts, such as *Colura irrorata* (Spruce) Heinrichs, Y.Yu, Schäf.-Verw. & Pócs [57] and *Myriocoleopsis* [58,59] support this hypothesis because these taxa are not only different in their ecological preference but are also distinct from their epiphyllous relatives in having creeping stolons, robust stems, and long androecial spikes. These characters are interpreted as adaptive characters for successful survival under running water. The observation of morphological distinctiveness of non-epiphyllous and epiphyllous species of *Colura* and *Myriocoleopsis* supports the argument that stabilizing selection acts on phenotypes of epiphyllous liverworts. Such case is congruent with the hypothesis of the breakdown of ecological constraints [60]. Thus, we assumed that the parallel evolution and/or phenotypic plasticity of theses adaptive traits may occur frequently in leafy liverworts occurring in extreme habitats in which only a limited number of strategies enable survival. In context of above indicated evidence, we hypothesized that cryptic species of the *Cololejunea lanciloba* complex are caused by strong stabilizing selection imposed by environmental conditions. 

Alternatively the limited morphological variation of *Cololejeunea* species may coincide with restricted developmental options provided by their rather simple body-plan [61]. Developmental constraints have been considered in bryophytes in the context of a disconnection between wide distribution of MTSs and molecular variation among populations [62]. Although we have insufficient knowledge of alternative functional constraints on phenotypic evolution of liverworts, habitat-driven morphological constraints may contribute to the occurrence of cryptic species in the *Cololejeunea lanciloba* complex. 

Polyploidization and/or hybridization are common causes of cryptic speciation in vascular plants [63,64] and mosses [65-68], but only a few cases of hybridization and/ or polyploidization have been reported in liverworts. These reports are distributed randomly through the phylogeny of liverworts and include some genera: *Ricca* [69], *Marchantia* [70], *Plagiochasma* [71], *Porella* [72] and *Pellia* [73], but did not include epiphyllous liverworts with the putative exception of *Trocholejeunea* [74]. The absence of evidence may be a result of the limited cytological studies or special life cycle period-haploid gametophyte. The latter hypothesis was consistent with current karyological observation on Lejeuneaceae including *Cololejeunea* [75-77]. Besides, our results did not provide evidence of hybridization and/or introgression because we did not observe any topological conflict between nuclear and plastid regions. However, we hesitate to make a conclusion of absence of hybridization and/or introgression based on utilization of only two loci. Future studies will hopefully explore this question using multiple loci because they can be effectively identified using next generation sequencing.

### DNA taxonomy and species delimitation of cryptic species

The significance of DNA sequences for the recognition of cryptic species has been widely accepted, since crossing experiments, considered as a final test for reproductive isolation [78], are impractical for the majority of organisms. This applies especially for organisms adapting to extreme habitats. However, DNA taxonomic approaches are still under development and require further empirical investigation, especially in the context of land plants [31,32,79]. The required number of loci to obtain a robust species hypothesis has been recognized as one of the main problems of DNA taxonomy [80]. Single-loci-based studies may result in misleading taxonomic conclusions because these analyses are likely more sensitive to processes such as incomplete lineage sorting, genetic drift and lateral transfer of genes through hybridization and introgression. Nevertheless, reliable estimates of species boundaries based on single or few loci were derived in simulation studies [33]. In the present study we used two of the most frequently sequenced loci in land plants: the nuclear region nrITS and the plastid region *trnL–F*. These loci are known to accumulate both inter- and intra-specific sequence divergence for liverworts lineages and provide information on two out of the three genomes of the plant cell, and the sequence evolution of these regions is widely regarded as neutral [81]. 

This sensitivity of P(AB) and P(RD) in species delimitation was also supported by our results (see Table 2). To our knowledge, this is the first study employing these criteria to delimit species in liverworts, although these or related criteria have already been used in several studies of animals [82,83]. Species recognition has been discussed in previous studies of liverworts using DNA data, but none of them introduced statistic criteria, instead of general criteria such as monophyly, paraphyly and genetic distance. Furthermore, our results supported the feasibility of the ‘tip-to-root’ approach [33] for species recognition. We also want to point out the possible recovery of morphological distinctiveness by exhaustive morphological studies such as geometric morphometrics [84], which may enable us to find morphological disparity in cryptic species. 

## Supporting Information

Figure S1 **Two maximum likelihood phylogenetic hypotheses obtained by separate analyses of nrITS (left) and *trnL-F* (right).** *Cololejeunea calcarea* was used as outgroup in both analyses. Bold branches have a bootstrap values ≥ 70%. Taxa names were given as defined by morphological species identification. Species numbers were given as in Table S1. To simplify the comparison, vertical bars indicated clades of specimens found in both analyses, whereas vertical lines were added to link the clades recognized in both analyses. Note the absence of convincing evidence for recombination.
(TIF)

Figure S2 **The haplotypes networks of MOTUs with multiple haplotypes, shown: Clade A, C, E, H and J.** These networks were constructed based on combined dataset of *trnL-F* and nrITS. Samples are numbered: 1. *C. planissima*, 2. *C. lanciloba*, 3. *C. latilobula*, and 4. *C. yakusimensis*. The haplotype with the highest frequency is displayed as a square, while other haplotypes are displayed as circles. The size of each circles and rectangle reflects the number of samples with a shared haplotype.
(TIF)

Figure S3 **Bayesian majority consensus tree based on the combined data set with a partition of cpDNA and nrDNA.** The insert shows the whole tree whereas the main part of the figure shows the detail of the part indicated by the grey box in the insert. Boxes indicate groups of species that were tested using species delimitation plugin implemented in Geneious and the results are shown in Table S3. The decoder for the “+” and “-” is as follows: P(RD)/P(AB)/Posterior probability (BP-PP). Significance was determined by: 0.05/10-5/0.95.
(PDF)

Table S1 **Names, origins, vouchers (herbarium) and Genbank accession numbers used for phylogenetic analyses in alphabetical order.** Sequences in bold were obtained from Genbank.
(DOCX)

Table S2 **Summary of the analyses of molecular operative taxonomic units (MOTUs) by the species delimitation plugin.** The MOTUs were defined by considering the result of the phylogenetic analyses (see Figure 1) and the results of statistics parsimony network analyses (SPNA). Only clades with bootstrap values ≥ 95% and posterior probability p ≥ 0.95 were considered. The numbers in column 1 are the number of individuals in each clade. The following values are shown: Intra-clade genetic distances (Intra Dist); the ratio of Intra-clade genetic distance to Inter-clade genetic distance (Intra/Inter); the mean probability, with a 95% confidence interval (CI) for a prediction of making a correct identification of an unknown specimen being found only in the group of interest (P ID (strict)); the mean probability, with a 95% confidence interval (CI) for a prediction of making a correct identification of an unknown specimen being sister to or within the group of interest (P ID (Liberal)); mean distance between the most recent common ancestor of the species and its members (Av(M A)) ; probability that a clade has the observed degree of distinctiveness P(Randomly Distinct, RD); Rosenberg’s reciprocal monophyly (P(AB)). Shaded numbers indicate genetically significance of MOUTs.
(DOCX)

Table S3 **Tip to root approach according to Boykin et al** [33] **applied on phylogeny of *Cololejeunea lanciloba* complex**.
Clade numbers refer to boxed individuals found in Figure S3. The information of each column was shown in Table S2. Shaded numbers indicate genetically significance of MOUTs.
(DOCX)
