# Supplementary figures and images for: Empirical Evidence Supporting Frequent Cryptic Speciation in Epiphyllous Liverworts: A Case Study of the Cololejeunea lanciloba Complex
Source: PLoS One. 2013 Dec 18;8(12):e84124. doi: 10.1371/journal.pone.0084124 (PMC3867491; doi:10.1371/journal.pone.0084124)

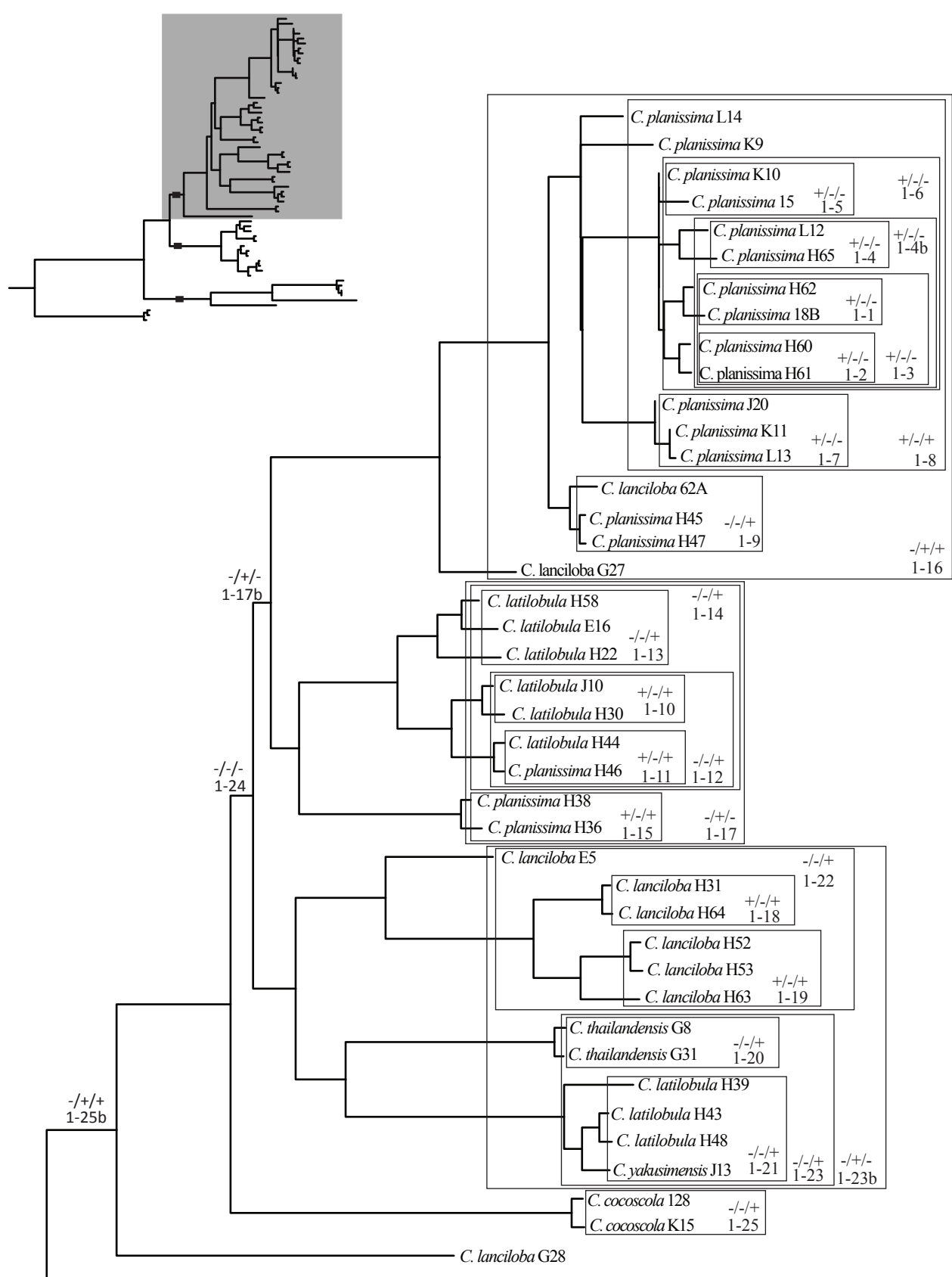

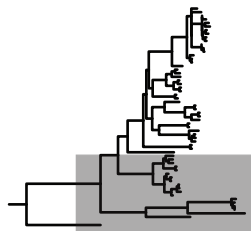

-/+/-  
1-25c

-/+/+  
1-25d

+ / + / +  
4-1

*C. calcarea* G35  
*C. calcarea* E6  
*C. calcarea* G36

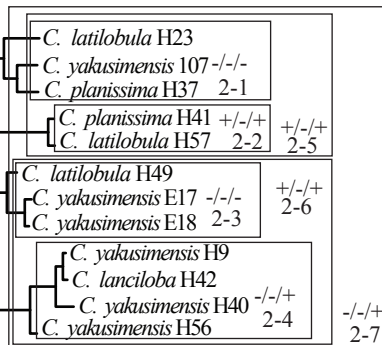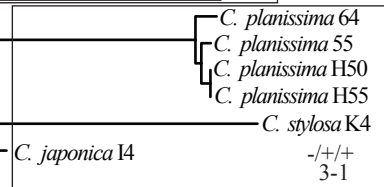

Supplement: Figure S3 — Bayesian majority consensus tree based on the combined data set with a partition of cpDNA and nrDNA. The insert shows the whole tree whereas the main part of the figure shows the detail of the part indicated by the grey box in the insert. Boxes indicate groups of species that were tested using species delimitation plugin implemented in Geneious and the results are shown in Table S3. The decoder for the “+” and “-” is as follows: P(RD)/P(AB)/Posterior probability (BP-PP). Significance was determined by: 0.05/10-5/0.95. (PDF) [file pone.0084124.s003.pdf]
