# Supplementary material for: Empirical Evidence Supporting Frequent Cryptic Speciation in Epiphyllous Liverworts: A Case Study of the Cololejeunea lanciloba Complex
Source: PLoS One. 2013 Dec 18;8(12):e84124. doi: 10.1371/journal.pone.0084124 (PMC3867491; doi:10.1371/journal.pone.0084124)
Supplement: Table S1 — Names, origins, vouchers (herbarium) and Genbank accession numbers used for phylogenetic analyses in alphabetical order. Sequences in bold were obtained from Genbank. (DOCX) [file pone.0084124.s004.docx]

**Table S1. Names, origins, vouchers (herbarium) and Genbank accession numbers used for phylogenetic analyses in alphabetical order.** Sequences in bold were obtained from Genbank.

| **Taxa** | **Ex.No.** | **Specimen (Herb.)** | **Origin** | ***trnL-F*** | **nrITS** |
| --- | --- | --- | --- | --- | --- |
| *Cololejeunea calcarea* (Libert.) Schiffn. | E6 | D.G.Long with E.Maier & A Caillau 38620 (E) | Switzerland | **JQ991275** | **JQ991045** |
| *Cololejeunea calcarea* | G35 | Schäfer-Verwimp & Verwimp 29744 (GOET) | Greece | **JQ991276** | **JQ991046** |
| *Cololejeunea calcarea* | G36 | Schäfer-Verwimp 31568 (GOET) | Germany | **JQ991277** | **JQ991047** |
| *Cololejeunea cocoscola* Tixier | 128 | Pócs & Pócs 03283/O (HSNU) | Fiji Islands | **JQ991282** | **JQ991052** |
| *Cololejeunea cocoscola* | K15 | Pócs & Pócs 03267/C (HSNU) | Fiji Islands | **JQ991283** | **JQ991053** |
| *Cololejeunea japonica* (Schiffn.) Mizut. | I4 | Zhu 20100421-21 (HSNU) | China, Zhejiang | **JQ991298** | **JQ991068** |
| *Cololejeunea lanciloba* Steph. | E5 | Long M2666h (E) | Malawi | JX843290 | JX843331 |
| *Cololejeunea lanciloba* | G27 | Sporn 91 (GOET) | Indonesia | **JQ991302** | **JQ991072** |
| *Cololejeunea lanciloba* | G28 | Pócs & Streimann 9960/T (GOET) | Australia | **JQ991303** | **JQ991073** |
| *Cololejeunea lanciloba* | H31 | Zhu *et al.* 20100821-19A (HSNU) | China, Guangxi | JX843291 | JX843332 |
| *Cololejeunea lanciloba* | H52 | Wei 20120205-7F (HSNU) | China, Guangxi | JX843292 | JX843333 |
| *Cololejeunea lanciloba* | H53 | Wei 20120205-6B (HSNU) | China, Guangxi | JX843293 | JX843334 |
| *Cololejeunea lanciloba* | H63 | Wei 20100221-40A (HSNU) | China, Guangxi | JX843294 | JX843335 |
| *Cololejeunea lanciloba* | H64 | Zhu *et al.* 20100821-20B (HSNU) | China, Guangxi | JX843295 | JX843336 |
| *Cololejeunea latilobula* (Herzog) Tixier | E16 | Long 28121 (E) | Bangladesh | **JQ991304** | **JQ991074** |
| *Cololejeunea latilobula* | H22 | Zhu 20111218-32 (HSNU) | Thailand | JX843296 | JX843337 |
| *Cololejeunea latilobula* | H23 | Wang & Peng 20110519-54A (HSNU) | China, Yunnan | JX843297 | JX843338 |
| *Cololejeunea latilobula* | H30 | Zhu et al. 20100827-41 (HSNU) | China, Guizhou | JX843298 | JX843339 |
| *Cololejeunea latilobula* | H39 | Wang & Peng 20111014-3A (HSNU) | China, Xizang | JX843299 | JX843340 |
| *Cololejeunea latilobula* | H40 | Wang & Peng 20111009-7 (HSNU) | China, Xizang | JX843300 | JX843341 |
| *Cololejeunea latilobula* | H43 | Wang & Peng 20110520-59 (HSNU) | China, Yunnan | JX843301 | JX843342 |
| *Cololejeunea latilobula* | H44 | Zhu & Wei 20120503-34 (HSNU) | China, Hongkong | JX843302 | JX843343 |
| *Cololejeunea latilobula* | H46 | Zhu & Wei 20120503-45 (HSNU) | China, Hongkong | JX843303 | JX843344 |
| *Cololejeunea latilobula* | H48 | Wang & Peng 20110520-6C (HSNU) | China, Yunnan | JX843304 | JX843345 |
| *Cololejeunea latilobula* | H49 | Wang & Peng 20110519-56A (HSNU) | China, Yunnan | JX843305 | JX843346 |
| *Cololejeunea latilobula* | H58 | Wang & Peng 20110513-1 (HSNU) | China, Yunnan | JX843306 | JX843347 |
| *Cololejeunea latilobula* | J10 | Peng & Wei 20100921-39 (HSNU) | China, Guangxi | **JQ991305** | **JQ991075** |
| *Cololejeunea latilobula* | J13 | Yu 20100921-1 (HSNU) | China, Yunnan | **JQ991366** | **JQ991136** |
| *Cololejeunea planissima* (Mitt.) Abeyw. | 55 | Zhu *et al.* 20100822-55 (HSNU) | China, Guangxi | **JQ991325** | **JQ991095** |
| *Cololejeunea planissima* | L12 | Peng & Wei (HSNU) | China, Guangxi | **JQ991328** | **JQ991098** |
| *Cololejeunea planissima* | 64 | Peng & Wei 20100912-64 (HSNU) | China, Guangxi | JX843308 | JX843349 |
| *Cololejeunea planissima* | H36 | Zhu 20111218-10 (HSNU) | Thailand | JX843309 | JX843350 |
| *Cololejeunea planissima* | H37 | Zhu 20111219-20E (HSNU) | Thailand | JX843310 | JX843351 |
| *Cololejeunea planissima* | H38 | Zhu 20111220-41 (HSNU) | Thailand | JX843311 | JX843352 |
| *Cololejeunea planissima* | H45 | Zhu & Wei 20120503-38 (HSNU) | China, Hongkong | JX843312 | JX843353 |
| *Cololejeunea planissima* | H47 | Zhu & Wei 20120503-11 (HSNU) | China, Hongkong | JX843313 | JX843354 |
| *Cololejeunea planissima* | H50 | Wei 20120204-15B (HSNU) | China, Guangxi | JX843314 | JX843355 |
| *Cololejeunea planissima* | H55 | Wei 20110214-9 (HSNU) | China, Guangxi | JX843315 | JX843356 |
| *Cololejeunea planissima* | H60 | Zhang *et al.* 20091031-15B (HSNU) | China, Fujian | JX843316 | JX843357 |
| *Cololejeunea planissima* | H61 | Zhang *et al.* 20091031-14B (HSNU) | China, Fujian | JX843317 | JX843358 |
| *Cololejeunea planissima* | H62 | Zhang *et al.* 20091031-17 (HSNU) | China, Fujian | JX843318 | JX843359 |
| *Cololejeunea planissima* | H65 | Zhang *et al.* 20100703-4 (HSNU) | China, Fujian | JX843319 | JX843360 |
| *Cololejeunea planissima* | J20 | Peng & Wei 20100915-38 (HSNU) | China, Guangxi | JX843320 | JX843361 |
| *Cololejeunea planissima* | K9 | Peng & Wei 20100915-62 (HSNU) | China, Guangxi | JX843323 | JX843364 |
| *Cololejeunea planissima* | K10 | Peng & Wei 20100912-50 (HSNU) | China, Guangxi | JX843321 | JX843362 |
| *Cololejeunea planissima* | K11 | Peng & Wei 20100912-40B (HSNU) | China, Guangxi | JX843322 | JX843363 |
| *Cololejeunea planissima* | L13 | Peng & Wei 20100915-36 (HSNU) | China, Guangxi | JX843324 | JX843365 |
| *Cololejeunea planissima* | L14 | Peng & Wei 20100915-46 (HSNU) | China, Guangxi | JX843325 | JX843366 |
| *Cololejeunea planissima* | L15 | Peng & Wei 20100916-20A (HSNU) | China, Guangxi | JX843326 | JX843367 |
| *Cololejeunea planissima* | 62A | Wei 20100210-62a (HSNU) | China, Guangxi | **JQ991301** | **JQ991071** |
| *Cololejeunea planissima* | 18B | Zhang *et al.* 20091031-18B (HSNU) | China, Fujian | JX843307 | JX843348 |
| *Cololejeunea stylosa* (Steph.) A. Evans | K4 | Peng & Yu (HSNU) | China, Hainan | **JQ991348** | **JQ991118** |
| *Cololejeunea thailandensis* Tixier | G31 | Schäfer-Verwimp & Verwimp 16233 (GOET) | Thailand | **JQ991353** | **JQ991123** |
| *Cololejeunea thailandensis* | G8 | Schäfer-Verwimp & Verwimp 16234/A (GOET) | Thailand | **JQ991354** | **JQ991124** |
| *Cololejeunea yakusimensis* (S.Hatto.)Mizut. | E17 | Long 34518A (E) | China, Yunnan | **JQ991326** | **JQ991096** |
| *Cololejeunea yakusimensis* | E18 | Long 34521 (E) | China, Yunnan | **JQ991327** | **JQ991097** |
| *Cololejeunea yakusimensis* | 107 | Yu 20100921-4 (HSNU) | China, Yunnan | **JQ991363** | **JQ991133** |
| *Cololejeunea yakusimensis* | H9 | Wang & Peng 20111018-86C (HSNU) | China, Xizang | **JQ991365** | **JQ991135** |
| *Cololejeunea yakusimensis* | H41 | Wang & Peng 20111018-91 (HSNU) | China, Xizang | JX843327 | JX843368 |
| *Cololejeunea yakusimensis* | H42 | Wang & Peng 20111018-65 (HSNU) | China, Xizang | JX843328 | JX843369 |
| *Cololejeunea yakusimensis* | H56 | Wang & Peng 20111018-94B (HSNU) | China, Xizang | JX843329 | JX843370 |
| *Cololejeunea yakusimensis* | H57 | Wang & Peng 20111009-3 (HSNU) | China, Xizang | JX843330 | JX843371 |
