# Supplementary material for: Empirical Evidence Supporting Frequent Cryptic Speciation in Epiphyllous Liverworts: A Case Study of the Cololejeunea lanciloba Complex
Source: PLoS One. 2013 Dec 18;8(12):e84124. doi: 10.1371/journal.pone.0084124 (PMC3867491; doi:10.1371/journal.pone.0084124)
Supplement: Table S2 — Summary of the analyses of molecular operative taxonomic units (MOTUs) by the species delimitation plugin. The MOTUs were defined by considering the result of the phylogenetic analyses (see Figure 1) and the results of statistics parsimony network analyses (SPNA). Only clades with bootstrap values ≥ 95% and posterior probability p ≥ 0.95 were considered. The numbers in column 1 are the number of individuals in each clade. The following values are shown: Intra-clade genetic distances (Intra Dist); the ratio of Intra-clade genetic distance to Inter-clade genetic distance (Intra/Inter); the mean probability, with a 95% confidence interval (CI) for a prediction of making a correct identification of an unknown specimen being found only in the group of interest (P ID (strict)); the mean probability, with a 95% confidence interval (CI) for a prediction of making a correct identification of an unknown specimen being sister to or within the group of interest (P ID (Liberal)); mean distance between the most recent common ancestor of the species and its members (Av(M A)) ; probability that a clade has the observed degree of distinctiveness P(Randomly Distinct, RD); Rosenberg’s reciprocal monophyly (P(AB)). Shaded numbers indicate genetically significance of MOUTs. (DOCX) [file pone.0084124.s005.docx]

**Table S2.** **Summary of the analyses of molecular operative taxonomic units (MOTUs) by the species delimitation plugin.** The MOTUs were defined by considering the result of the phylogenetic analyses (see Fig. 1) and the results of statistics parsimony network analyses (SPNA). Only clades with bootstrap values ≥ 95% and posterior porability p ≥ 0.95 were considered. The numbers in column 1 are the number of individuals in each clade. The following values are shown: Intra-clade genetic distances (Intra Dist); the ratio of Intra-clade genetic distance to Inter-clade genetic distance (Intra/Inter); the mean probability, with a 95% confidence interval (CI) for a prediction of making a correct identification of an unknown specimen being found only in the group of interest (P ID (strict)); the mean probability, with a 95% confidence interval (CI) for a prediction of making a correct identification of an unknown specimen being sister to or within the group of interest (P ID (Liberal)); mean distance between the most recent common ancestor of the species and its members (Av(M A)) ; probability that a clade has the observed degree of distinctiveness P(Randomly Distinct, RD); Rosenberg’s reciprocal monophyly (P (AB)). Shaded numbers indicate genetically significance of MOUTs.

| MOTUs | Individuals | Intra Dist | intra/inter | PID(strict) | PID (Liberal) | Av(MRCA-tips) | P(RD) | P(AB) |
| --- | --- | --- | --- | --- | --- | --- | --- | --- |
| Clade A | 17 | 0.247 | 0.16 | 0.94(0.89,0.99) | 0.98(0.95, 1.0) | 0.3297 | 1 | 2.50E-08 |
| Clade B | 2 |  |  |  |  |  |  |  |
| Clade C | 7 | 0.229 | 0.4 | 0.75(0.64,0.85) | 0.91(0.84,0.97) | 0.1543 | 0.61 | 0.01 |
| Clade D | 2 | 4.70E-02 | 0.08 | 0.55(0.40,0.70) | 0.93(0.78,1.0) | 0.0236 | <0.05 | 0.01 |
| Clade E | 5 | 0.214 | 0.23 | 0.78(0.65,0.90) | 0.95(0.85,1.0) | 0.1471 | 1 | 0.07 |
| Clade F | 1 |  |  |  |  |  |  |  |
| Clade G | 2 |  |  |  |  |  |  |  |
| Clade H | 4 | 0.123 | 0.16 | 0.76(0.62,0.90) | 0.94(0.83,1.0) | 0.0797 | 1 | 0.03 |
| Clade I | 1 |  |  |  |  |  |  |  |
| Clade J | 12 | 0.338 | 0.23 | 0.90(0.83,0.97) | 0.97(0.92,1.0) | 0.243 | 0.86 | 1.40E-13 |
| Clade K | 4 | 0.059 | 0.03 | 0.85(0.71,0.99) | 0.98(0.87,1.0) | 0.0405 | <0.05 | 0.1 |
