# Supplementary material for: Empirical Evidence Supporting Frequent Cryptic Speciation in Epiphyllous Liverworts: A Case Study of the Cololejeunea lanciloba Complex
Source: PLoS One. 2013 Dec 18;8(12):e84124. doi: 10.1371/journal.pone.0084124 (PMC3867491; doi:10.1371/journal.pone.0084124)
Supplement: Table S3 — Tip to root approach according to Boykin et al [33] applied on phylogeny of Cololejeunea lanciloba complex. Clade numbers refer to boxed individuals found in Figure S3. The information of each column was shown in Table S2. Shaded numbers indicate genetically significance of MOUTs. (DOCX) [file pone.0084124.s006.docx]

**Table S3. Tip to root approach according to Boykin et al. [25] applied on phylogeny of *C.lanciloba* complex.** Clade numbers refer to boxed individuals found in Figure S3. The information of each column was shown in Table S2. Shaded numbers indicate genetically significance of MOUTs.

| Clade 1 | Clade 2 | Intra Dist | intra/inter | PID(strict) | PID (Liberal) | Av(MRCA-tips) | P(RD) | P(AB) | BP-PP |
| --- | --- | --- | --- | --- | --- | --- | --- | --- | --- |
| 1-1(2) | 1-2(2) | 0.047 | 0.49 | 0.34(0.18,0.49) | 0.68(0.52,0.83) | 0.0234 | <0.05 | 0.11 | 0.16 |
| 1-2 | 1-1 | 0.035 | 0.37 | 0.40(0.25,0.55) | 0.75(0.60,0.91) | 0.0176 | <0.05 | 0.11 | 0.22 |
| 1-3(4) | 1-4(2) | 0.077 | 0.56 | 0.49(0.34,0.64) | 0.79(0.68,0.90) | 0.0474 | <0.05 | 0.03 | 0.80 |
| 1-4 | 1-3 | 0.100 | 0.73 | 0.22(0.06,0.37) | 0.53(0.37,0.69) | 0.0502 | <0.05 | 0.03 | 0.91 |
| 1-4b(6) | 1-5 | 0.111 | 1.27 | 0.08(00E+00) | 0.36(0.258,0.46) | 0.0642 | <0.05 | 0.01 | 0.09 |
| 1-5 | 1-4b | 0.045 | 0.52 | 0.32(0.17,0.48) | 0.66(0.51,0.82) | 0.0227 | <0.05 | 0.01 | 0.06 |
| 1-6(8) | 1-7(3) | 0.098 | 0.33 | 0.79(0.68,0.89) | 0.92(0.86,0.98) | 0.0538 | <0.05 | 1.21E-0.3 | 0.01 |
| 1-7 | 1-6 | 0.021 | 0.07 | 0.74(0.57,0.92) | 0.97(0.82,1.0) | 0.0182 | <0.05 | 1.21E-03 | 0.02 |
| 1-8(13) | 1-9(3) | 0.202 | 0.77 | 0.68(0.62,0.75) | 0.90(0.86,0.94) | 0.1514 | <0.05 | 2.3E-4 | 1.0 |
| 1-9 | 1-8 | 0.050 | 0.19 | 0.67(0.49,0.84) | 0.90(0.76,1.0) | 0.0299 | 0.83 | 2.3E-4 | 0.97 |
| 1-10(2) | 1-11(2) | 0.051 | 0.33 | 0.42(0.27,0.58) | 0.78(0.63,0.93) | 0.0253 | 0.05 | 0.11 | 1.0 |
| 1-11 | 1-10 | 0.032 | 0.21 | 0.48(0.33,0.64) | 0.85(0.70,1.0) | 0.0161 | <0.05 | 0.11 | 1.0 |
| 1-12(4) | 1-13(3) | 0.116 | 0.38 | 0.61(0.47,0.76) | 0.87(0.76,0.98) | 0.0769 | 0.44 | 0.01 | 1.0 |
| 1-13 | 1-12 | 0.145 | 0.47 | 0.48(0.30,0.66) | 0.74(0.59,0.89) | 0.0848 | 0.38 | 0.01 | 1.0 |
| 1-14(7) | 1-15(2) | 0.229 | 0.40 | 0.75(0.64,0.85) | 0.91(0.84,0.97) | 0.1543 | 0.61 | 0.01 | 1.0 |
| 1-15 | 1-14 | 0.047 | 0.08 | 0.55(0.40,0.70) | 0.93(0.78,1.0) | 0.0236 | <0.05 | 0.01 | 1.0 |
| 1-16(17) | 1-17(9) | 0.247 | 0.26 | 0.91(0.85,0.96) | 0.97(0.94,1.00) | 0.3297 | 1.00 | 2.5E-8 | 1.0 |
| 1-17 | 1-16 | 0.360 | 0.39 | 0.78(0.70,0.87) | 0.93(0.88,0.98) | 0.2986 | 0.75 | 2.5E-8 | 0.76 |
| 1-18(2) | 1-19(2) | 0.028 | 0.10 | 0.54(0.39,0.69) | 0.92(0.77,1.0) | 0.0141 | <0.05 | 0.05 | 1.0 |
| 1-19 | 1-18 | 0.134 | 0.47 | 0.48(0.30,0.66) | 0.74(0.59,0.89) | 0.0927 | <0.05 | 0.05 | 0.99 |
| 1-20(2) | 1-21(4) | 0.031 | 0.04 | 0.57(0.42,0.72) | 0.96(0.81,1.0) | 0.0156 | 0.44 | 0.03 | 1.0 |
| 1-21 | 1-20 | 0.123 | 0.16 | 0.76(0.62,0.90) | 0.94(0.83,1.0) | 0.0797 | 1.00 | 0.03 | 1.0 |
| 1-22(6) | 1-23(6) | 0.323 | 0.34 | 0.70(0.58,0.83) | 0.92(0.82,1.0) | 0.3401 | 0.95 | 1.9E-4 | 1.0 |
| 1-23 | 1-22 | 0.453 | 0.48 | 0.61(0.49,0.74) | 0.87(0.77,0.97) | 0.3900 | 0.84 | 1.9E-4 | 0.99 |
| 1-17b(26) | 1-23b(12) | 0.582 | 0.54 | 0.83(0.78,0.88) | 0.96(0.93,0.98) | 0.5040 | 0.72 | 1.9E-11 | 0.29 |
| 1-23b(12) | 1-17b(26) | 0.692 | 0.65 | 0.75(0.68,0.81) | 0.93(0.88,0.97) | 0.4729 | 0.79 | 1.9E-11 | 0.82 |
| 1-24(38) | 1-25(2) | 0.809 | 0.73 | 0.76(0.71,0.81) | 0.93(0.91,0.96) | 0.5336 | 0.96 | 6.5E-5 | 0.74 |
| 1-25 | 1-24 | 0.037 | 0.03 | 0.57(0.42,0.72) | 0.96(0.81,1.0) | 0.0183 | 1.00 | 6.5E-5 | 1.0 |
| 2-1(3) | 2-2(2) | 0.146 | 0.57 | 0.41(0.23,0.59) | 0.69(0.54,0.83) | 0.0838 | 0.57 | 0.05 | 0.85 |
| 2-2 | 2-1 | 0.030 | 0.12 | 0.53(0.38,0.68) | 0.91(0.76,1.0) | 0.0152 | <0.05 | 0.05 | 1.0 |
| 2-3(3) | 2-4(4) | 0.063 | 0.29 | 0.60(0.42,0.78) | 0.84(0.70,0.99) | 0.0461 | 0.51 | 0.01 | 0.58 |
| 2-4 | 2-3 | 0.082 | 0.37 | 0.62(0.47,0.76) | 0.87(0.77,0.98) | 0.0753 | 0.48 | 0.01 | 1.0 |
| 2-5(5) | 2-6(7) | 0.201 | 0.41 | 0.66(0.53,0.78) | 0.90(0.80,1.00) | 0.1247 | <0.05 | 2.2E-4 | 1.0 |
| 2-6 | 2-5 | 0.159 | 0.33 | 0.79(0.68,0.89) | 0.92(0.86,0.98) | 0.1165 | <0.05 | 2.2E-4 | 1.0 |
| 1-25b(41) | 2-7(12) | 0.858 | 0.58 | 0.82(0.77,0.87) | 0.95(0.93,0.98) | 0.7373 | 0.61 | 1.4E-13 | 1.0 |
| 2-7 | 1-25b | 0.338 | 0.23 | 0.90(0.83,0.97) | 0.97(0.92,1.0) | 0.2430 | 0.86 | 1.4E-13 | 1.0 |
| 1-25c(53) | 3-1(4) | 1.055 | 0.43 | 0.86(0.81,0.92) | 0.96(0.93,0.99) | 0.7974 | 0.53 | 7.6E-10 | 0.82 |
| 3-1 | 1-25c | 0.843 | 0.35 | 0.70(0.58,0.83) | 0.92(0.82,1.0) | 0.9339 | 0.93 | 7.6E-10 | 1.0 |
| 1-25d(59) | 4-1(3) | 1.310 | 0.48 | 0.85(0.80,0.90) | 0.96(0.93,0.99) | 1.0396 | 0.93 | 8.6E-7 | 1.0 |
| 4-1 | 1-25d | 0.052 | 0.02 | 0.78(0.60,0.95) | 1.00(0.85,1.0) | 0.0349 | <0.05 | 8.6E-7 | 1.0 |
